# Non-Pharmacological Pain Treatment of Patients with Myofascial Pain Syndrome of the Masticatory Muscles—Case Series

**DOI:** 10.3390/biomedicines11102799

**Published:** 2023-10-16

**Authors:** Monica Macrì, Chiara Rotelli, Francesco Pegreffi, Felice Festa

**Affiliations:** 1 Department of Innovative Technologies in Medicine & Dentistry, University “G. D’Annunzio” of Chieti-Pescara, 66100 Chieti, Italy; 2 Department for Life Quality Studies, University of Bologna, 40126 Bologna, Italy

**Keywords:** myofascial pain, temporomandibular joint, temporomandibular disorder, TMD, TMJ MRI

## Abstract

Myofascial pain is the most common cause of chronic pain in the masticatory region and can be assessed through clinical analysis and muscle palpation. Generally, it appears with headache and orofacial pain associated with sensitive points (trigger points) due to the excessive contraction of the masticatory muscle fibers. The study aims to evaluate how a correct treatment of myofascial pain can improve the life quality of affected patients. In this case series, 300 patients with myofascial pain were divided into two groups: 150 with intra- and extra-articular disorders and 150 with only extra-articular disorder. Each group included 75 males and 75 females. All the patients were treated with gnathological therapy through passive aligners and biofeedback exercises for four months. They underwent pain assessment (through a visual analogue scale and muscular palpation test) before, during, and after the treatment, as well as nuclear magnetic resonance of the temporomandibular joint before and after the gnathological treatment. The treatment considerably reduced the pain in all patients, without drugs, in four months according to the visual analogue scales and the palpation test. The temporomandibular magnetic resonance in each patient was similar before and after the gnathological treatment. The improvement in pain did not depend on a change in the relationship between the articular condyle and the disc.

## 1. Introduction

Temporomandibular disorders (TMDs) are a group of musculoskeletal and neuromuscular conditions involving the temporomandibular joint complex and the surrounding musculature and osseous components. TMDs affect up to 15% of adults, with a peak incidence in patients between 20 and 40 years of age. 

In the context of the multifactorial etiology of TMDs, the importance of the psychological component has been demonstrated.

Chronic stress alters the body’s physiological processes, causing inflammation, oxidative stress, and changes in hormones and immune function, increasing the risk of developing chronic diseases. For example, a correlation between TMDs and post-traumatic disorder in war veterans has also been found; war is one of the primary sources of exposure to stress [1]. Some studies have demonstrated a greater incidence of TMDs due to the recent COVID-19 pandemic [2].

A systematic review demonstrated the high prevalence of depressive disorders in patients with TMDs [3]. A correlation between psychosocial disorders and TMDs was found in this case [4]. TMDs are also often associated with sleep disorders, anxiety, and depression in a two-way relationship since psychosomatic disorders can be both a cause and a consequence of TMDs. Psychological stress can alter and induce painful sensations and contribute to the development of TMDs [5]. It has also been shown that improving the outcome of sleep disorders also enhances TMD symptoms [6].

TMDs are classified as intra-articular and extra-articular diseases. Intra-articular TMDs are congenital, developmental, or degenerative disorders in which the relationship between the articular disc, the mandibular condyle, and the glenoid fossa is impaired. Extra-articular TMDs include disorders involving the masticatory muscles such as local myalgia, myofascial pain disorder, myofibrotic contracture, myositis, and myospasm [7]. The etiology of TMDs is multifactorial. Temporomandibular disorders have a wide range of causes, among which the most common are severe malocclusions, stress, anxiety, mandibular instability, postural imbalance, pathological conditions, and parafunctional habits [8,9,10]. Parafunctional habits, such as bruxism and clenching, cause constant micro trauma in the temporomandibular joint, inducing pain in the affected areas [11]. Myofascial pain syndrome is the most prevalent condition associated with unconscious clenching [12] and is usually chronic. In the new DC/TMD, the term “myofascial pain” implies two diagnoses: myofascial pain (pain spreading beyond the site of palpation but within the palpated muscle) and myofascial pain with referral (pain of an area beyond the boundary of the palpated muscle) [13]. 

This painful condition is characterized by myofascial trigger points (MTrPs) [14], which are hyperirritable spots that provoke hyperalgesia and pain when compressed and can lead to orofacial pain, headache, and motor dysfunction. This pain can also radiate to distant sites [15]. Two types of myofascial trigger points (MTrPs) can be distinguished: active MTrPs and latent MTrPs. An active TrP produces constant pain, while a latent TrP causes pain only during palpation [16]. The scientific evidence of the remarkable sensory capacity of muscle fasciae can be explained through this review. It investigates histological and immunohistochemical aspects of fascial innervation, suggesting that the fasciae participate actively in proprioception and nociception, constituting the largest sensory organ of our body. Considering the innervation density between the masseter muscle and its fascia, it was demonstrated that the latter is more innerved (404.5 fibers/area mm^2^ inside the connective tissue) than the muscle (227.6 fibers/area mm^2^). Moreover, most of the nerves were nociceptors [17]. Pathological fascia is characterized by increased tissue stiffness and alterations in myofibroblast activity and the extra-cellular matrix in terms of collagen and matrix metalloprotease (MMP) levels. Pain originating from the deep fascia likely results from increased nerve density, sensitization, and chronic nociceptive physical or chemical stimulation [18]. 

Studies also reported that tension-type headaches (TTHs) and migraines are associated with referred pain from TrPs in several muscular areas such as the suboccipital, upper trapezius, sternocleidomastoid, temporalis, or superior oblique muscles. Referred pain caused by active TrPs simulates the pain areas detected during head pain episodes in these primary headaches [19,20,21]. One interesting theory that underlines the importance of MTrPs and their relationship to headaches is that the progression from episodic to chronic forms of TTHs is related to prolonged nociceptive input from peripheral myofascial tissues [22].

Primary headache disorders are unrelated to an underlying medical condition and are classified into four groups: migraines, tension-type headaches, trigeminal autonomic cephalalgias, and other primary headaches. Secondary headache disorders are related to an underlying medical condition.

Migraine is the most debilitating primary headache disorder and affects 12% of the population. It is characterized by several symptoms, with headaches as the hallmark. It can be treated acutely with analgesics, nonsteroidal anti-inflammatory drugs, triptans, gepants, and lasmiditan [23]. Migraines can be classified according to frequency and subdivided into migraines with or without aura.

A migraine attack can last 4–72 h and consists of four overlapping stages.

(a) Premonitory phase: This happens hours or days before the onset of the headache, with non-painful symptoms such as frequent yawning, difficulty concentrating, fatigue, and thirst.
(b) Aura: This is a transient focal neurological symptom that can happen before or during some of the headaches. Visual aura is the most frequent type (90%), followed by sensory (30–54%), and language auras (31%).
(c) Headache: The result of the activation of the trigeminal sensory pathways, which generates the throbbing pain. It interferes with daily activities and typically gets worse with head movement. It is usually associated with nausea, vomiting, and aversion to light (photophobia) and sound (phonophobia).
(d) Postdrome: Characterized by tiredness, drowsiness, difficulty concentrating, and hypersensitivity to noise [24].

Myofascial trigger points are prevalent in migraine and tension-type headaches, but their role in the pathophysiology of each disorder and to what degree is unclarified [25].

Myofascial pain in masticatory muscles is associated with parafunctional habits such as teeth clenching and grinding and emotional factors such as distress. Parafunctional patterns during the day and sleep bruxism induce the intensification of muscle tension connected to myofascial pain [26]. The muscle symptoms include fatigue and increased tension, especially in elevator muscles (m. Masseter and m. Temporalis) [27,28]. Patients refer to pain in specific areas, especially around the jaw, temples, and ears. 

The presence of myofascial trigger points and associated orofacial and referred pain diagnose myofascial pain syndrome. The trigger points can be detected by palpation of the muscles, which consists of palpating the muscle perpendicular to the direction of the muscle fibers [29]. The palpation pressure is 1 kg for 2 s. To differentiate the types of myalgia, the duration of this pressure is increased up to 5 s to elicit spreading or referred pain if present [30]. 

Furthermore, ultrasound imaging makes assessing the muscles and fasciae of the head and neck region activity and the trigger point areas possible. Ultrasound permits the evaluation of the ability of the muscles to contract and identifies functional asymmetry that could become symptomatic [31]. Ultrasound images can help diagnose temporomandibular dysfunction, but cases, for example, that are scheduled for surgery should be evaluated with MRI [32].

This study aimed to evaluate reductions in head and orofacial pain caused by MTrPs associated with unconscious teeth clenching. Patients were treated with passive aligners and biofeedback exercises to teach them not to clench their teeth [33]. The treatment lasted four months. 

Due to this important correlation between chronic pain disorders and the psychosomatic state of patients, we were very interested in alternative and non-pharmacological management of the symptoms of myofascial pain syndrome.

An adequate, non-pharmacological, gnathological therapy leads to the remission of orofacial pain and active trigger points [34,35].

## 2. Materials and Methods

This case series was conducted in the Oral Sciences Department of the University of Chieti G. D’Annunzio. The study protocol was designed per the European Union Good Practice Rules and the Helsinki Declaration. Each patient provided written informed consent to participate in this study. Ethics approval (number 23) was obtained by the hospital’s Independent Ethics Committee of Chieti. 

The study considered diagnosing myofascial pain with referral headaches according to DC/TDM and ICHD-3 beta criteria. 

All 300 selected patients underwent a quantitative pain assessment through a visual analogue scale (VAS) and palpation test before and during treatment and magnetic resonance imaging of the temporomandibular joint (TMJ MRI) before and after the gnathological treatment. The initial TMJ MRI was required to collocate patients in the right groups; the final TMJ MRI was needed to assess the unaltered relationship between the disc and condyle.

The study lasted for three years (2020–2022): eighteen months to recruit the patients, twelve months for the follow-up, and six months for the data processing. The patients were treated for four months. 

Sample size 

358 patients were eligible for the study [36]. 42 declined to participate and 16 were lost during follow-up. 

The sample included 300 patients with unconscious teeth clenching and myofascial pain syndrome. 

150 patients with extra-articular TMDs were included in the first group, whereas 150 patients with extra and intraarticular TMDs were included in the second group. 

Each group comprised 75 men and 75 women. 

Inclusion Criteria

(1) Patients between 18 and 55 years of age.
(2) Diagnosis of myofascial pain in the masticatory muscles.
(3) Pain, including headache, in the last 30 days since the stated sensitivity (new DC/TMD)
(4) Average pain severity of four on a ten-point scale for at least 1 h daily.

Exclusion Criteria

(1) Pregnancy.
(2) Psychiatric disorder or current use of psychiatric medications.
(3) The presence of the cause of the chronic pain disorder.
(4) Family history of arthritis or gout.
(5) Current use of non-steroidal anti-inflammatory drugs, paracetamol, or opioids.

Measurements

VAS: visual analogue scale. A graphic representation of a patient’s face where they had to highlight painful areas, specifying the intensity (from 0 = No Pain to 10 = Maximum Pain) and occurrence of the disturbance [37]. 

Each patient completed the VAS before and after treatment and each month in the follow-up appointments. Every month, the same operator visited each patient.

PALPATION: The aim was to find trigger points in masticatory muscles (temporal, masseter, sternocleidomastoid, digastric, and pterygoid muscles), which, once stimulated, produce or increase orofacial pain and referred headache. 

The same operator performed the palpation on all patients every month. The operator manually exerted a pressure of 1 kg bilaterally for 5 s on the masseter, temporal, pterygoid, sternocleidomastoid, and digastric masticatory muscles. 

The pain was classified by the patient on a scale from zero to three:

- 0: absence of pain.
- 1: mild pain or apparent discomfort.
- 2: moderate pain or discomfort.
- 3: severe pain [38].

Each patient underwent a palpation test before and after treatment and each month in the follow-up appointments. Every month, the same operator performed the palpation test. 

MRI TMJ: MRI evaluated the integrity of the temporomandibular joint. 

It was used to diagnose intra-articular or extra-articular disorders before treatment, to localize patients in the correct group, and to assess changes in the condyle–disc relationship after treatment. 

Each patient underwent a TMJ MRI with open-mouth and closed-mouth postures before and after the treatment. 

Treatment protocol

The treatment protocol used two passive Essix splints (lower and upper). To obtain the greatest possible comfort for the patient, the splints were made of polycarbonate and customized in the mouth to avoid irritation or damage to the soft tissues. The thickness of the splints was <0.7 mm. 

The patients wore the lower passive aligner splint (LPAS) during the daytime and the upper passive aligner splint (UPAS) at night. The splint was removed only at mealtimes and during oral hygiene, and the two splints were never in the mouth simultaneously.

While wearing the LPAS, the patients performed a biofeedback exercise for two minutes daily (before breakfast, lunch, and dinner) over the study duration to enable us to manage the masticatory muscle activity.

To avoid bias during the biofeedback exercise, the patients had to maintain an upright or lying position without crossing their legs or arms. Traditionally, biofeedback is presented to the patient and the clinician via visual displays and acoustic or vibrotactile feedback [39]. In our case, the patient was asked to imagine a tennis ball during the exercise phases, so the neuronal activation during the biofeedback visualization corresponded to the action phase.

The exercise consisted of four steps [40]. 

The total contraction on the masseters and the teeth clenching characterized the first step. The patients had to visualize the maximum contraction of the muscles as a fully inflated tennis ball, with the help of a light touch of the cheeks with the forefinger.

In the second step, the patients had to clench their teeth, partially contracting the masseter (on each side) and associating a light touch with the forefinger. A semi-deflated tennis ball was visualized this time [40].

During the third step, the patients fully relaxed their jaw (opened about 1 mm). They applied a light touch with the forefinger to the fully relaxed masseter, visualizing the muscle’s volume as a deflated tennis ball.

In the last step, the patients touched the top of the palatine vault with the tip of their tongue for five seconds [40].

The duration of the treatment (and consequently of the performance of the biofeedback exercise) was four months. Then, a new assessment was made using the VAS and palpation of the pertinent muscles, and TMJ MRI was repeated to evaluate the treatment effect and outcome.

The patients recorded pain diaries during the study period to supervise compliance and examine and monitor symptom development [40]. 

Patients were followed up after one year, and only six of them showed relapses with mild symptoms [40]. 

Study Protocol

The subject selection required the diagnosis of myofascial pain syndrome based on a standardized and complete clinical examination, which fulfilled the Research Diagnostic Criteria (RDC TMDs) [41]. 

After recruitment, they underwent a TMJ MRI to assess the intra-articular TMJ condition. 

The study included 300 patients with myofascial pain matched for sex, 150 with intra-articular and extra-articular TMDs and 150 with extra-articular TMDs, classified by the TMJ MRI.

In the study’s first phase, the patients underwent the palpation test and visual analogue scale to assess, measure, and locate the pain. 

In the study’s second phase, the patients were treated with the gnathological therapy previously described for four months. All patients underwent follow-up appointments once a month, in which they underwent the VAS and palpation test again with the same operator every time. 

After four months, TMJ MRIs with open-mouth and closed-mouth postures were performed again. 

Statistical methods

All statistical analysis was performed using SPSS 26.0 (IBM, Armonk, NY, USA) and evaluated at a two-tailed alpha level of 0.05. The sample population was assessed to determine the deviation ratio for women to men from the expected 1:1 (Fisher’s exact test) and differences in the distribution of VAS scores (Pearson’s chi-squared). The effect of the treatment protocol on modifying the parameters was assessed using a Wilcoxon signed ranks test. Correlations between the modification of VAS with the baseline and changes in palpation measurements were evaluated using Spearman’s rho. 

## 3. Results

All patients were affected by orofacial pain and headache but no migraine. 

Nine out of the three hundred included patients were excluded from the study because they did not follow the operator’s instructions about using splints and biofeedback exercises. 

After treatment, the primary outcome was pain reduction, assessed using the VAS and palpation test after four months. Patients were re-examined after one year; only six showed relapses with mild orofacial pain. Nevertheless, these patients did not require any medications. 

We observed that pain symptomatology decreased in all patients by comparing the baseline values (T1) to post-treatment values (T2). The improvements were unrelated to age and gender but related to symptom intensity and chronicity at T1. 

The characteristics of the study group are reported in Table 1. 

All patients completed the treatment protocol using gnathological therapy consisting of passive aligners [42] and biofeedback exercises, and nine patients were lost during follow-up. 

The statistical results are reported in Table 2. 

The sample population did not differ from the expected 1:1 ratio of women to men, and the distribution of VAS scores with age did not differ between the sexes. Treatment significantly modified the VAS and palpation scores (*p* < 0.001, Wilcoxon signed ranks test). 

The modification of VAS scores was highly correlated (positively) with the change in the masseter palpation score (*p* = 0.001) and correlated with the improvement in the sternocleidomastoid palpation score (*p* = 0.016). There was a trend toward statistical significance with the pterygoid palpation score (*p* = 0.092). The modifications in palpation scores were all highly correlated, with a range of *p* < 0.001 (to *p* = 0.021), except for temporal–pterygoid, masseter–pterygoid, and pterygoid–digastric correlations. 

Initial palpitation scores did not correlate with modifications in the VAS score, except for the pterygoid palpation score (*p* = 0.030, positive correlation). 

When dividing the sample population into women and men separately, only the initial temporal palpation scores negatively correlated with the modification of the VAS score (*p* = 0.040).

The impact of the gnathological treatment on trigger points (palpation) in the 150 patients with the extra-articular disorders and the 150 patients with the intra-ed extra-articular disorder was assessed by comparing the baseline (T1) and post-treatment (T2) pain extents, and intensity of pain during palpation test decreased uniformly. Again, the improvements were not related to age and gender but to symptom intensity and chronicity at T1. 

## 4. Discussion

This study reports the impact of gnathological therapies with passive aligners [42] and biofeedback exercises in patients with myofascial pain syndrome associated with unconscious teeth clenching [42]. All patients had orofacial pain and headache as referred pain of MTrPs. 

Myofascial pain can be treated through invasive or non-invasive procedures. Non-invasive procedures include spray-and-stretch, transcutaneous electrical stimulation, physical therapy, and massage. Among them, fascial manipulation was shown to reduce pain and is the gold standard in orofacial pain treatment [43]. Invasive treatments for myofascial trigger points include injections with local anesthetics, corticosteroids, botulinum toxin, or dry needling [44]. Most patients improve with a combination of non-invasive therapies such as patient education, self-care, cognitive behavior therapy, pharmacotherapy, physical therapy, and occlusal devices. Some patients are also treated with nonsteroidal anti-inflammatory drugs and muscle relaxants; benzodiazepines or antidepressants may be added for chronic cases. Oral and maxillofacial surgery is indicated for refractory cases [45].

The biofeedback technique is a method to teach patients to recognize, correct, and prevent the physiological alterations underlying various pathological conditions, with their consequent reduction or elimination. 

Biofeedback approaches to headache therapy fall into two broad categories: general biofeedback techniques and techniques more directly related to the pathophysiology underlying the onset of the headache. Several meta-analyses have evaluated the use of available biofeedback-assisted relaxation techniques for headaches. These reviews indicate that various biofeedback techniques are effective for migraine and tension-type headaches [46]. Biofeedback exercises of the tongue aim at enhancing patients’ awareness about the position of palatal arches associated with jaw clenching so that they can learn to stop or decrease this habit. 

Although some studies have demonstrated the effectiveness of biofeedback in neuromuscular system physical rehabilitation [47], our protocol used muscle biofeedback associated with passive splints to best control the unconscious nocturnal and daily clenching.

This study included 300 pts (150 men and 150 women), aged between 18 and 55 years, who showed orofacial pain (VAS values between 5 and 10), masticatory muscle trigger points, and mandible movement restriction. None of the 300 patients experienced migraines and none had a family history of arthritis or gout. 

After the treatment, we noticed a significant (Table 1 and Table 2) decrease in the symptomatology, in terms of both referred pain (VAS) and trigger points (previously detected by muscle palpation). The TMJ resonance did not change the disc–condyle relationship in patients with extra- and intra-articular disorders or those with only extra-articular disorders. However, the treatment effectively reduced pain regardless of the intra-articular relation between the condyle and disc. All patients showed a significant decrease in the symptoms after four months of therapy and did not report any relapse after one year. 

The treatment protocol does not change the intra-articular interaction between the disc and condyle, as revealed by the MR analysis of the TMJ, thus limiting its action to ameliorating the symptoms [48]. However, joint noises do not necessarily correlate with pain severity or functional limitation. The most appropriate markers of treatment success are the absence of pain, improved function, and standard quality of life [48]. A lack of clicking is not a reliable indicator of whether a patient has responded to treatment [49].

Finally, our findings demonstrate that this therapeutical setup efficiently reduces [50] myofascial syndrome pain in intra- and extra-articular TMD patients, regardless of age and gender [50]. It is of great interest that the pain is reduced without pharmacological treatment or dental orthodontic or prosthetic treatments. The muscle biofeedback exercise [51] is sufficient to teach the patient not to clench their teeth and the splints to release the arches [51]. The muscle relaxation obtained implies a reduction in pain and improved function.

We consider these results very important, especially because they occurred after the COVID-19 pandemic, during which there has been a serious increase in stress and, consequently, in the prevalence of TMDs. Furthermore, this data is very interesting as it enables chronic pain management without using drugs. Because the use of medications during the pandemic has been chaotic and controversial, patients have greatly appreciated the resolution of pain without a medication prescription, improving their quality of life and mood.

No improvements have been observed in patients with intra-articular disorder regarding disc dislocation. Pain relief does not depend on a change in intra-articular condition.

## 5. Conclusions

From our perspective, the importance of this research lies in the fact that orofacial pain was treated with a conservative non-medical treatment that requires only four months. The patients were re-examined after one year, and only six showed a mild relapse, which did not require any drugs.

The limit of this study is the wide range of ages. Nevertheless, we think it can be helpful to those clinicians who want to treat orofacial pain and referred headaches caused by myofascial pain syndrome of the masticatory muscles without medications and with a low relapse rate after one year.

## Figures and Tables

**Table 1:** Study population characteristics.

	Women	Men	Total
N	141 (48%)	150 (52%)	291
Age	36.2 + 10.4	36.7 + 10.5	36.5 + 10.5
VAS T0	7.9 + 1.3	8 + 1.1	8 + 1.2
VAS T1	1.4 + 1.2	1.2 + 1.2	1.4 + 1.3
Delta VAS	6.5 + 1.4	6.7 + 1.2	6.7 + 1.4
Masseter T0	2.5 + 0.5	2.6 + 0.4	2.6 + 0.5
Masseter T1	0.5 + 0.6	0.5 + 0.5	0.6 + 0.6
Temporal T0	2 + 0.6	2.1 + 0.6	2.1 + 0.7
Temporal T1	0.3 + 0.4	0.3 + 0.4	0.4 + 0.5
Pterygoid T0	2.8 + 0.3	2.8 + 0.3	2.9 + 0.4
Pterygoid T1	0.8 + 0.6	0.8 + 0.6	0.9 + 0.7
Sternocleidomastoid T0	1.8 + 0.8	1.8 + 0.8	1.9 + 0.8
Sternocleidomastoid T1	0.2 + 0.4	0.2 + 0.4	0.3 + 0.5
Digastric T0	0.8 + 0.7	0.9 + 0.7	0.9 + 0.8
Digastric T1	0 + 0.1	0 + 0.1	0 + 0.2
Delta Masseter	2 + 0.6	2.1 + 0.6	2.1 + 0.6
Delta Temporal	1.7 + 0.6	1.7 + 0.6	1.7 + 0.6
Delta Pterygoid	1.9 + 0.6	1.9 + 0.6	2 + 0.7
Delta Sternocleidomastoid	1.5 + 0.7	1.6 + 0.7	1.6 + 0.8
Delta Digastric	0.8 + 0.7	0.8 + 0.7	0.9 + 0.7

T0: time zero. T1: time one. VAS: visual analogue scale.

**Table 2:** Statistical results.

VAS with		Masseter	Temporal	Pterygoid	Sternocleidomastoid	Digastric
T0						
Women	CC	0.124	−0.087	0.061	−0.027	0.160
	p	0.143	0.304	0.473	0.752	0.059
Men	CC	0.047	−0.168	0.032	−0.008	−0.116
	p	0.565	0.040 *	0.696	0.918	0.157
						
delta						
Women	CC	0.124	−0.087	0.061	−0.027	0.160
	p	0.143	0.304	0.473	0.752	0.059
Men	CC	0.114	−0.143	0.069	0.071	−0.096
	p	0.166	0.082	0.401	0.386	0.241

* T0: time zero.

## Data Availability

Data available on request due to restrictions.
